# 

**Published:** 1842-07-02

**Authors:** 


**CONTENTS OF No. 27.**

Dr. P. J. Bauduy's cases of Tetanus successfully treated, - - - - 417
Dr. Wilson's Clinical Reports - - - - - - - - 426
Pericarditis - - - - - - 426
Pleuritis - - - - - - - 429
ANALECTA: Dr. R. W. Scott, on Bronchorrhœa Æstiva, or Hay-Fever, 430
Abscess of the Iliac Fossa, - - - - - - - 432

